# A Phenotypic Study of *CRB1* Retinopathy Secondary to the Variant p.(Pro836Thr) Prevalent in Those of Black African Ancestry

**DOI:** 10.1167/iovs.66.9.3

**Published:** 2025-07-01

**Authors:** Wendy M. Wong, Anthony G. Robson, Rebecca A. Baker, Gavin Arno, Joseph Van Aerschot, Siying Lin, Mariya Moosajee, Michel Michaelides, Omar A. Mahroo, Andrew R. Webster

**Affiliations:** 1 Moorfields Eye Hospital NHS Foundation Trust, London, United Kingdom; 2 Centre for Innovation and Precision Eye Health, Yong Loo Lin School of Medicine, National University of Singapore, Singapore; 3 UCL Institute of Ophthalmology, London, United Kingdom; 4 J. C. Self Research Institute, Greenwood Genetic Center, Greenwood, South Carolina, United States; 5 Department of Ophthalmology, University Hospitals Leuven, Leuven, Belgium; 6 Manchester Centre for Genomic Medicine, Saint Mary's Hospital, Manchester University NHS Foundation Trust, Manchester, United Kingdom; 7 Division of Evolution, Infection and Genomics, School of Biological Sciences, Faculty of Biology, Medicine and Health, University of Manchester, Manchester, United Kingdom

**Keywords:** *CRB1*, electrophysiology, genetics, inherited retinal disease, phenotype

## Abstract

**Purpose:**

To comprehensively characterize the clinical consequences of the *CRB1* variant p.(Pro836Thr). In African populations, this variant has an allele frequency of 0.329% (gnomAD v4.1.0).

**Methods:**

This study was a retrospective case series of 14 patients from 11 families with molecularly confirmed *CRB1*-associated retinal dystrophy, each possessing at least one p.(Pro836Thr) variant. The age at onset of visual symptoms, best-corrected visual acuity, imaging findings, and quantitative electrophysiologic measurements of retinal function were analyzed.

**Results:**

The p.(Pro836Thr) variant was homozygous in four families and compound heterozygous in seven families. The familial origins included Nigeria (*n* = 4), Ghana (*n* = 3), the Caribbean region (*n* = 2), and Uganda (*n* = 1). The median follow-up was 7 years (interquartile range, 3–16). Symptom onset was most common in childhood (eight patients, 57.1%). Reduced central vision was the most frequent presenting symptom (12 patients, 85%). Widefield multimodal imaging revealed peripheral retinal changes in addition to macular changes in three patients. Nine patients had international standard electrophysiology and showed generalized retinal dysfunction with a similar degree of rod and cone system involvement (*n* = 7) or a clear rod–cone pattern of dysfunction (*n* = 2). All had pattern electroretinography (ERG) evidence of macular dysfunction.

**Conclusions:**

The study highlights the association of the p.(Pro836Thr) variant with African ancestry and characterizes their key clinical and electrophysiological features. Our study suggests that the p.(Pro836Thr) variant confers a less severe consequence on retinal function and structure than the majority of other reported *CRB1* variants. Although retinal imaging may show alterations confined to the macular region, electrophysiology in this series indicates generalized cone and rod photoreceptor dysfunction.

Crumbs (Crb), first discovered in *Drosophila*, is an evolutionarily conserved protein that recruits other cell polarity regulators to form the Crb complex, which is essential for retinal development and regulation of cell adhesion.^1^^–^^4^ The Crumbs homolog 1 (*CRB1*) gene is expressed in the brain and the retina, where it localizes to the microvilli of Müller glial cells and inner segments of photoreceptors.^5^^,^^6^ Biallelic disease-causing variants in *CRB1* (OMIM *60420) are associated with a broad spectrum of inherited retinal disease (IRD) phenotypes: (1) Leber congenital amaurosis (LCA); (2) early-onset severe retinal dystrophy; (3) rod–cone dystrophy, which can occur in conjunction with preserved para-arteriolar retinal pigment epithelium (PPRPE) and/or a Coats-like exudative vasculopathy; (4) cone–rod dystrophy; (5) macular dystrophy; (6) foveal retinoschisis; and (7) fenestrated slit maculopathy.^7^^–^^12^

The *CRB1* gene spans 210 kb of genomic DNA on chromosome 1q31.3 and consists of 12 exons.^6^^,^^13^ Thus far, mRNA corresponding to three *CRB1* transcripts has been identified in the retina.^6^^,^^14^ The canonical 1406-aa isoform (*CRB1-A*, GenBank accession number MT470365) is most abundant during development; it has a large extracellular domain containing multiple epidermal growth factor–like domains and three laminin A globular (AG)-like domains (Fig. 1), as well as a transmembrane domain and highly conserved 37-aa intracellular domain.^6^^,^^9^^,^^15^ A second validated transcript is predominant in the adult retina and encodes a shorter 1003-aa protein (*CRB1-B*, GenBank accession number MT470366).^15^^,^^16^ The third validated transcript encodes a 754-aa isoform (*CRB1-C*, GenBank accession number MT470367) without transmembrane and intracellular domains.^6^^,^^15^ More than 450 variants have been linked to *CRB1*-associated retinal dystrophies, with the majority clustering in exons 7 (27%) and 9 (41%), which encode the second and the third laminin AG-like domains.^11^^,^^16^

**Figure 1.**
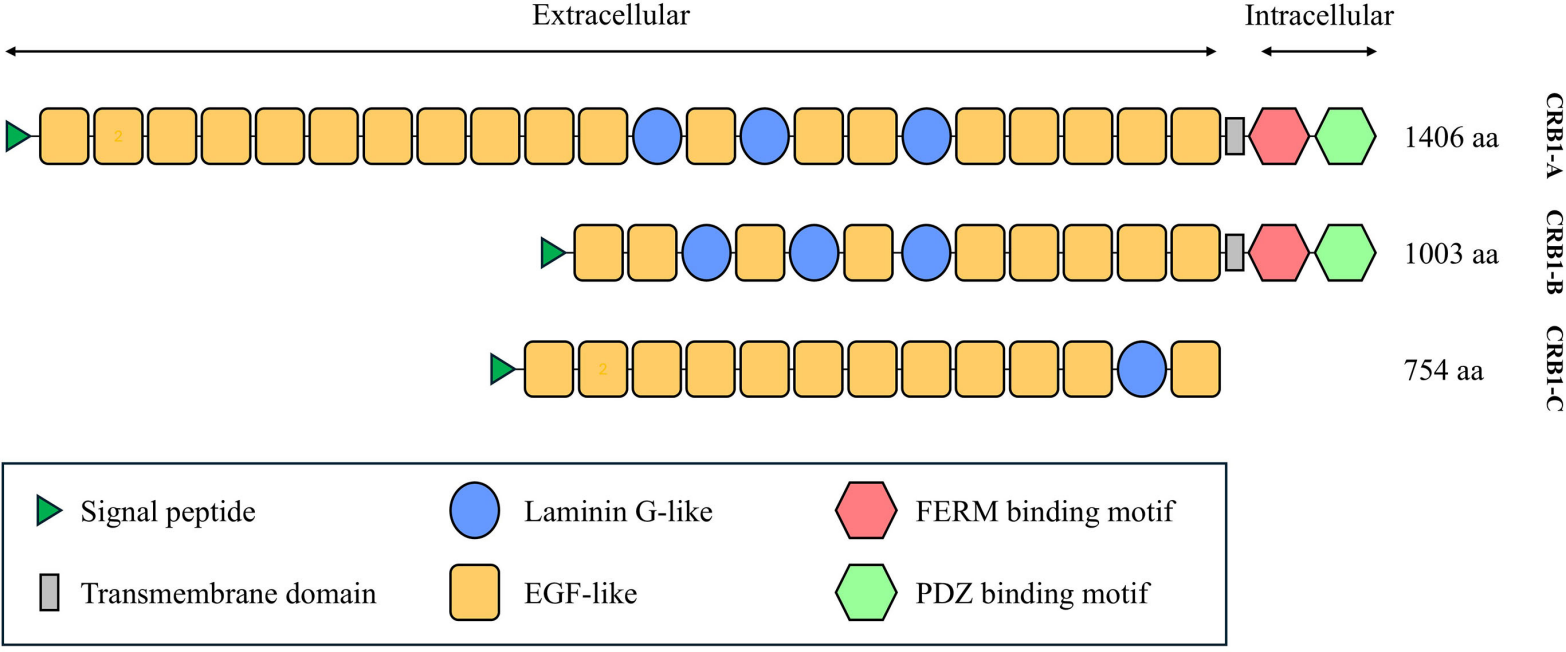
Schematic representation of the domains present in *CRB1-A* (GenBank version MT470365.1), *CRB1-B* (GenBank version MT470366.1), and *CRB1-C* isoforms (GenBank version MT470367.1).

A growing number of molecularly diagnosed IRD cohorts have revealed that several *CRB1* variants have a relatively high prevalence in specific ethnic populations.^11^^,^^17^^,^^18^ For example, the in-frame deletion p.(Ile167_Gly169del) has an overall population allele frequency of 0.123%, which rises to 0.156% in European populations (gnomAD v4.1.0).^17^ In a Chinese cohort, the p.(Gly1226Ter) variant is especially prevalent.^18^ In African populations the p.(Pro836Thr) variant has a high allele frequency of 0.329% (gnomAD v4.1.0), and was recently recognized as a cause of *CRB1* retinopathy in four families.^19^ This study aims to comprehensively characterize the clinical consequence of the *CRB1* variant p.(Pro836Thr).

## Methods

This retrospective study was approved by the local ethics committee (Moorfields Eye Hospital and the Northwest London Research Ethics Committee) and adhered to the tenets of the Declaration of Helsinki. Patients were identified using an in-house database (OpenEyes, London, UK) and only included patients with molecularly confirmed *CRB1*-associated retinal dystrophy with at least one (NM_201253.3) c.2506C>A; p.(Pro836Thr) variant. The molecular diagnosis was established using next-generation sequencing (panel testing of retinal dystrophy genes, whole exome sequencing, or whole genome sequencing). The sequence variants were interpreted according to the guidelines from the American College of Medical Genetics and Genomics and the Association for Molecular Pathology.^20^ Population allele frequencies were derived from gnomAD v4.1.0.

The phenotype was determined through a comprehensive review of the clinical history, multimodal imaging and available functional assessments from electrophysiological testing. Relevant clinical information extracted from the electronic medical records included: (1) demographics, (2) genetic information, (3) age at onset, (4) presenting symptoms, (5) family history, and (6) best-corrected Snellen visual acuity at the time of presentation. In patients reviewed from 2014 onward, the Optos confocal scanning laser ophthalmoscopy system (Optos, Dunfermline, UK) was used to acquire ultra-widefield (UWF) pseudocolor fundus photographs and UWF green wavelength autofluorescence; the SPECTRALIS system (Heidelberg Engineering, Heidelberg, Germany) was used to acquire spectral-domain optical coherence tomography (OCT) scans.

Pattern electroretinography (PERG) and full-field ERG were performed to incorporate the International Society for Clinical Electrophysiology of Vision (ISCEV) standards in nine individuals (age range, 8–50 years), using gold foil corneal recording electrodes.^21^^,^^22^ The two youngest (both 5 years old) were tested using skin electrodes according to abbreviated protocols, including one without mydriasis tested using non-Ganzfeld flash stimulation.^23^ PERG P50 was used as a measure of macular function, and the full-field ERG was used to assess generalized (mainly peripheral) rod and cone system function. The main components of the ISCEV dark-adapted (DA) and light-adapted (LA) ERGs were quantified and compared with age-matched control data from healthy subjects (age range, 10–79 years), with ERG amplitudes plotted as a percentage of the age-matched lower limit of the reference range and peak times plotted as a difference from the age-matched upper limit of the reference range.^24^^,^^25^

## Results

Fourteen individuals (seven females, seven males) from 11 families were identified. The familial origins included Nigeria (four pedigrees), Ghana (three pedigrees), the Caribbean region (two pedigrees), and Uganda (one pedigree). The p.(Pro836Thr) variant was homozygous in four families (Table). In seven families that were compound heterozygous for the p.(Pro836Thr) variant, the second variant was a missense in five families, a nonsense variant in one family, and a deletion of exon 6 in one family. The amino acid residues predicted to be affected are illustrated in Figure 1.

**Table.** Clinical Summary

	Allele 2				At Presentation and at Final Review			Structural Imaging			
Family(ID), Familial Origins, and Sex	Exon	Nucleotide and Protein Change	Presenting Symptoms	Age at Onset (y)	Age (y)	VA OD	VA OS	Affected Areas on FP and AF	Cystic Spaces on OCT	Initially Suspected Phenotype Clinically	Full-FieldERG Phenotype(Case No. According to Fig. 4)
F1(I)	7	c.2506C>A	Decreased vision	Child-hood	41	6/12	6/12	Macula and peripheral retina	Present OU	Retinal dystrophy	Cone and rod dystrophy (case 1)
Ghana		p.(Pro836Thr)			61	6/36	6/36				
Female											
F1(II)			Decreased night vision	43	51	6/12	6/18	Not available	Present OU	Cone–rod dystrophy	Cone and rod dystrophy (case 2)
Ghana			then central vision		53	6/12	6/12				
Male											
F2(I)	7	c.2506C>A	Decreased vision	21	27	6/12	6/9	Macula only	Present OU	Macular dystrophy	N/A
Nigeria		p.(Pro836Thr)			30	6/12	6/12				
Female											
F3(I)	2	c.470G>C	Decreased	Child-hood	6	6/36	6/36	Macula and nasal to OD	Absent	Cone–rod dystrophy	Cone and rod dystrophy (case 3)
St Lucia and France		p.(Cys157Ser)	vision		34	6/60	6/60				
Male											
F4(I)	7	c.2506C>A	Decreased vision	40	43	3/60	6/12	Macula and nasal to OD	Absent	Cone–rod dystrophy	Cone and rod dystrophy (case 4)
Nigeria		p.(Pro836Thr)			46	1/60	6/18				
Female											
F5(I)	7	c.2506C>A	Decreased central and night vision	40s	68	CF	CF	Macula and nasal to OD	Absent	Cone–rod dystrophy	N/A
Jamaica		p.(Pro836Thr)									
Male											
F6(I)	2	c.601T>C	Decreased vision	5	9	6/15	6/15	Macula only	Present OU	Foveal schisis	N/A
Ghana		p.(Cys201Arg)			12	6/12	6/12				
Male											
F7(I)	6	c.1183G>T	Decreased vision	6	7	6/48	6/48	Macula and nasal to OD	`Present OU	Foveal schisis	Cone–rod dystrophy (pediatric protocol with skin electrodes)
Nigeria and Zimbabwe		p.(Glu395Ter)			14	6/30	6/30				
Male											
F8(I)	6	Deletion of exon 6	Decreased vision	23	23	6/60	6/60	Macula and nasal to OD	Absent	Retinal dystrophy	Cone and rod dystrophy (case 5)
Ghana					39	CF	6/60				
Female											
F9(I)	7	c.2234C>T	Decreased vision	6	6	6/38	6/38	Macula only	Absent	Macular dystrophy	Mild cone dystrophy (pediatric protocol with skin electrodes)
Unknown		p.(Thr745Met)			13	6/38	6/48				
Male											
F10(I)	7	c.2676G>C	Decreased vision	15	15	6/15	6/15	Macula only	Absent	Macular dystrophy	Cone and rod dystrophy (case 6)
Nigeria		p.(Lys892Asn)			31	6/95	6/75				
Female											
F10(II)			Decreased	9	9	6/9	6/9	Macula and peripheral retina	Absent	Retinal dystrophy	Cone and rod dystrophy (case 7)
Nigeria			vision		24	6/60	6/36				
Female											
F10(III)			Decreased vision	Child-hood	18	6/12	6/18	Macula only	Present OD	Retinal dystrophy	Rod–cone dystrophy (case 8)
Nigeria					19	6/12	6/12				
Male											
F11(I)	3	c.716G>A	Decreased	12	33	6/24	6/24	Macula and peripheral retina	Present OU	Retinal dystrophy	Rod–cone dystrophy (case 9)
Uganda		p.(Cys239Tyr)	vision		56	6/60	3/60				
Female											

AF, autofluorescence; CF, counting fingers; ERG, electroretinogram; FP, fundus photography; OCT, optical coherence tomography; OD, right eye; OS, left eye; OU, both eyes; VA, visual acuity (Snellen); N/A, not available.

**Figure 2.**
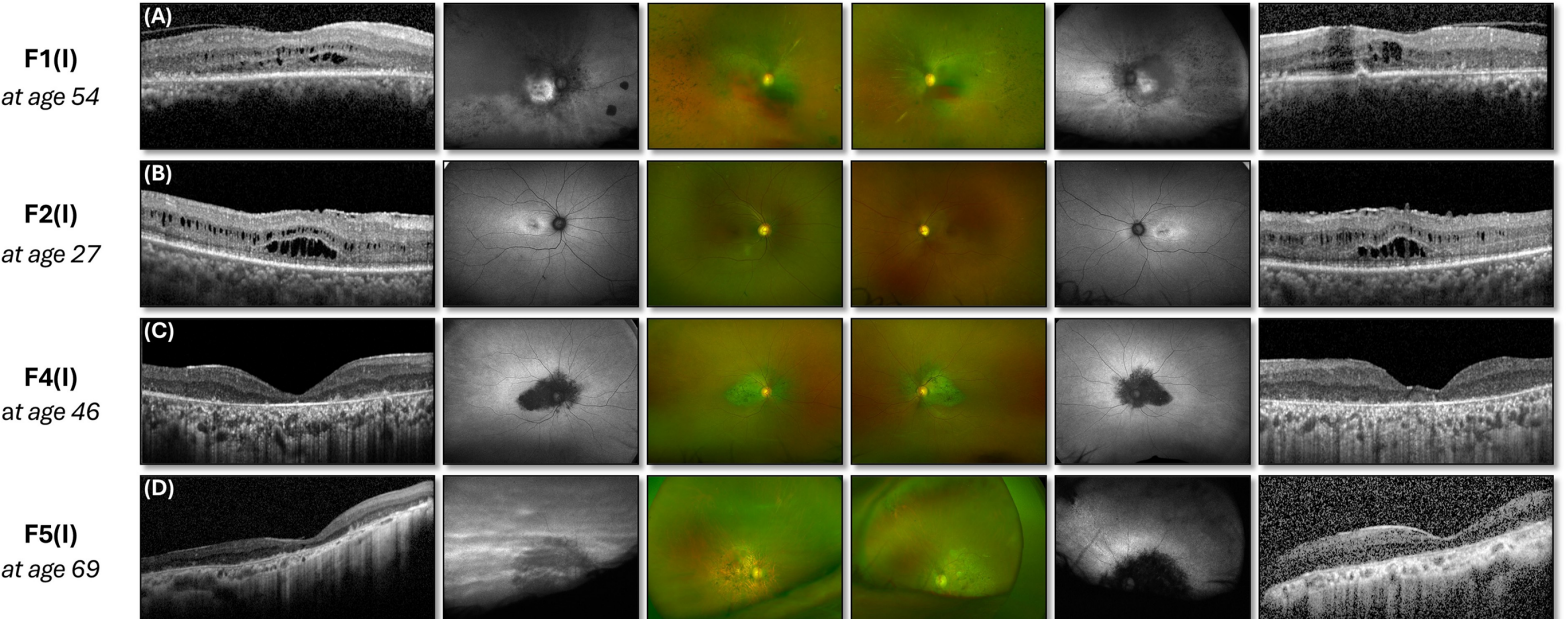
Multimodal imaging of patients homozygous for the p.(Pro836Thr) variant. (**A**–**D**) The optical coherence tomography scans of all patients show an abnormally laminated and thickened retina. Macular cystic spaces, when present, affected both the inner and outer nuclear layers (**A**, **B**).

**Figure 3.**
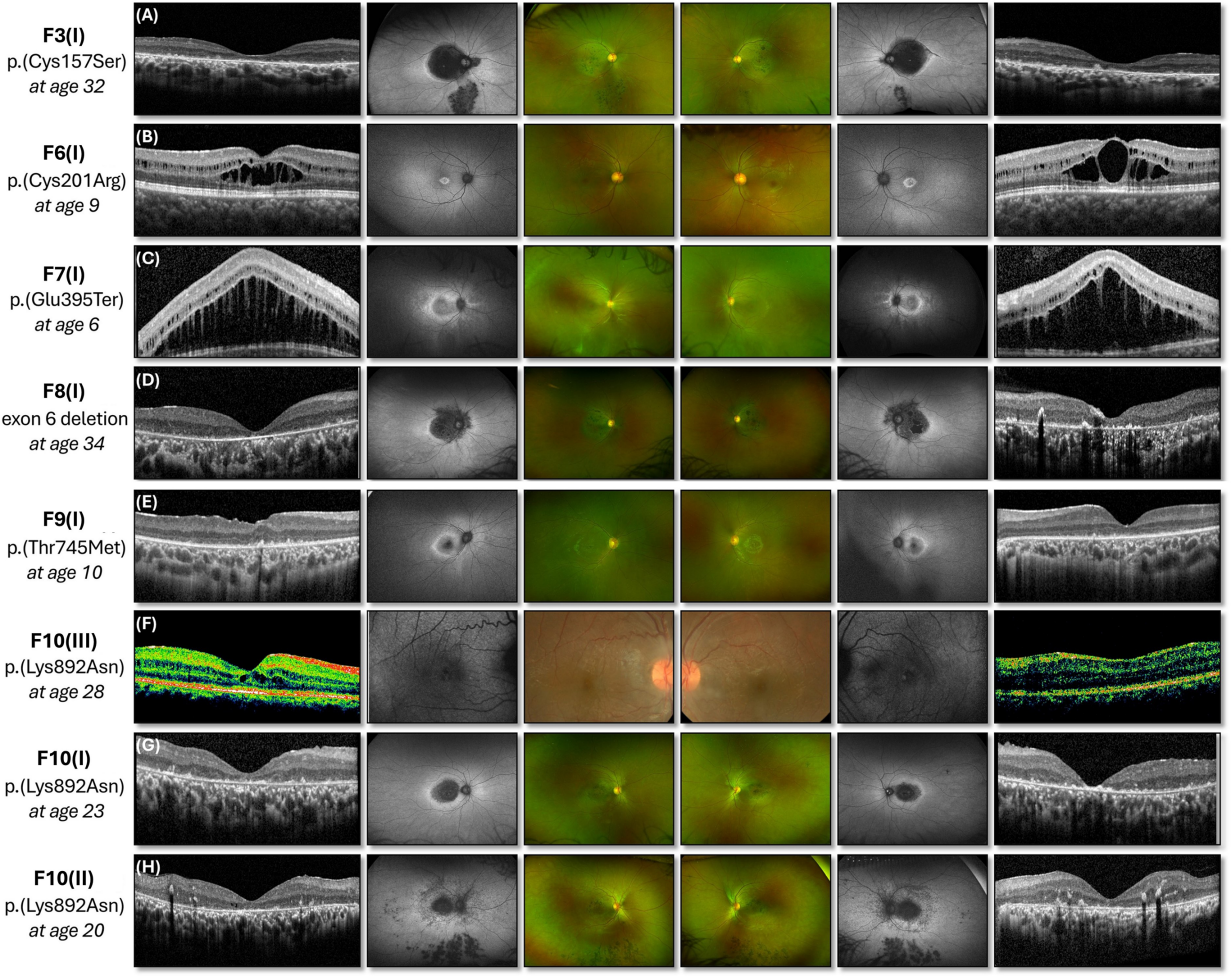
Multimodal imaging of patients who are compound heterozygous for the p.(Pro836Thr) variant (**A**–**H**). Marked intrafamilial phenotypic variation between three siblings was observed in F11; the youngest sibling has the earliest symptom onset, and additional involvement of the peripheral retina was evident on widefield fundus imaging.

Symptom onset was most commonly in mid- to late childhood (eight patients, 57.1%), although three patients did not observe symptoms until their fourth decade (21.4%). Reduced central vision was the most frequent presenting symptom (12 patients, 85%). In two patients, nyctalopia either preceded or coincided with the observation of reduced central vision. Nystagmus was present only in one patient, G15465(I). All patients had reduced visual acuity (range, 6/9 to counting fingers) and bilateral disease at presentation (Figs. 2, 3). The median follow-up was 7 years (interquartile range [IQR], 3–16). At the last follow-up, the median age was 31 years (IQR, 19–46), and deterioration in visual acuity was documented in nine patients (64.3%).

Nummular pigmentary changes in the macular region were visible in six patients on UWF fundus photography (magnified view of the central retina in Supplementary Fig. S1), with corresponding UWF autofluorescence demonstrating confluent hypo-autofluorescence involving the fovea, parafovea, and perifovea. The hypo-autofluorescence extended nasal to the optic disc in all but one case (Supplementary Fig. S1). Intraretinal migration of retinal pigment epithelium (RPE) cells was visible as intraretinal hyper-reflective foci on OCT and could be identified within both the outer and inner retinal layers (Figs. 3D, 3H). Mid-peripheral bone-spicule–like pigmentary deposits were observed in three patients (Figs. 2A, 3A, 3H), with PPRPE evident only in a single patient (Fig. 3H).

Electrophysiology testing was performed in 11 patients. The ISCEV standard full-field ERGs showed a high degree of interocular symmetry based on amplitudes of the DA 0.01, DA 3, and DA 10 ERG a-waves and b-waves; LA 30-Hz ERG and LA 3 ERG b-waves (slope = 0.95; *r*^2^ = 0.96); and on the peak times of the DA 3 and DA 10 ERG a-waves and b-waves and LA 30-Hz and LA 3 ERGs (slope = 1.05; *r*^2^ = 0.98).

**Figure 4.**
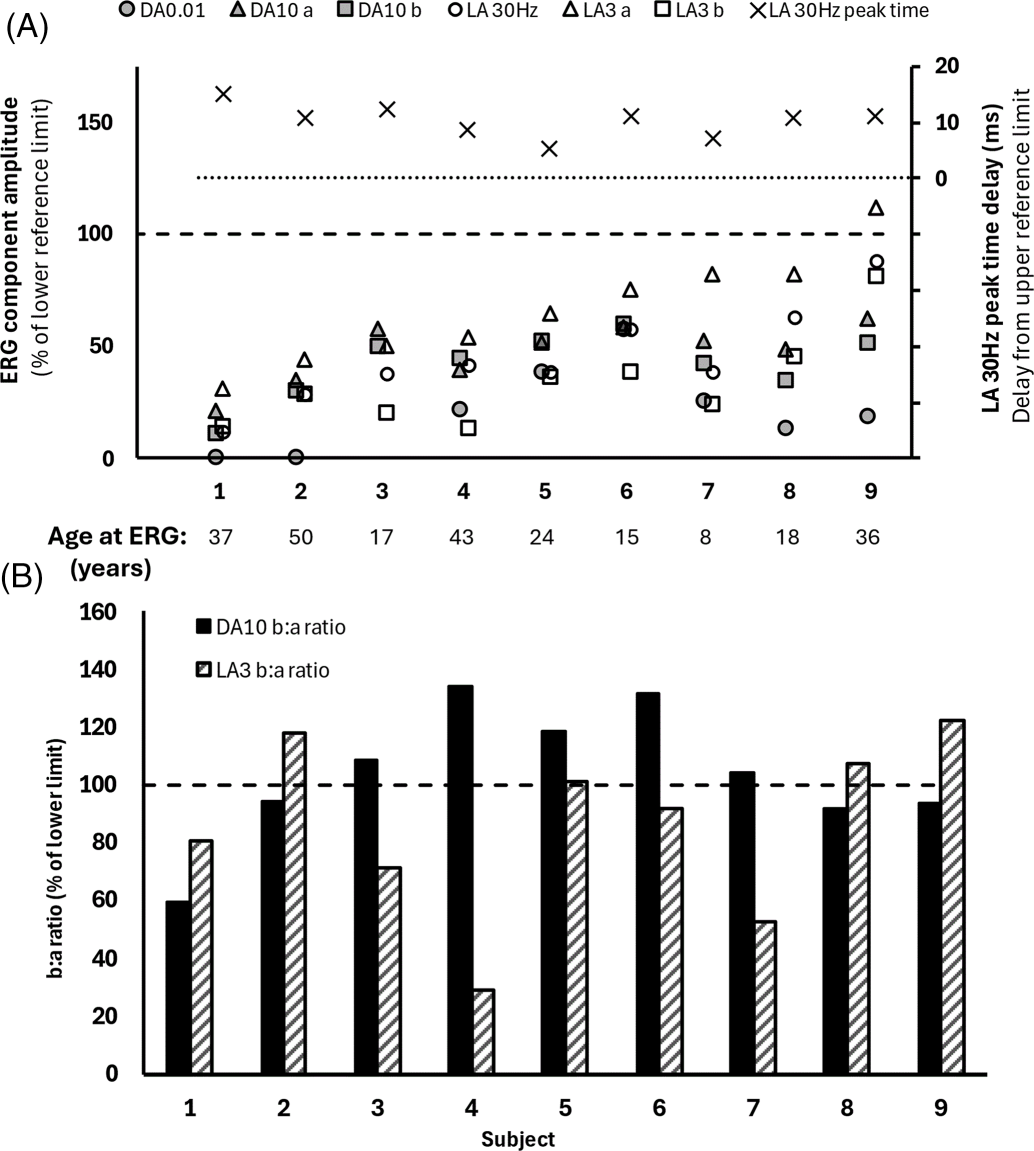
Full-field findings in subjects who were tested according to ISCEV standard methods. (**A**) The amplitudes of the DA 0.01 ERG, DA 10 ERG a-waves and b- waves, and LA 30-Hz ERG and LA 3 ERG a-waves and b- waves are plotted as a percentage of the age-matched lower limit of the reference (“normal”) range, with values arranged in ascending order of LA 3 ERG a-waves for clarity (primary *y*-axis). The LA 30-Hz peak times are plotted as a difference from the age-matched upper limit of the reference range (secondary *y*-axis). All electrophysiological recordings showed a high degree of interocular symmetry and are shown for right eyes only. (**B**) The DA 10 ERG and LA 3 ERG b:a ratios are compared with the lower limit of the reference range and are shown as a percentage.

**Figure 5.**
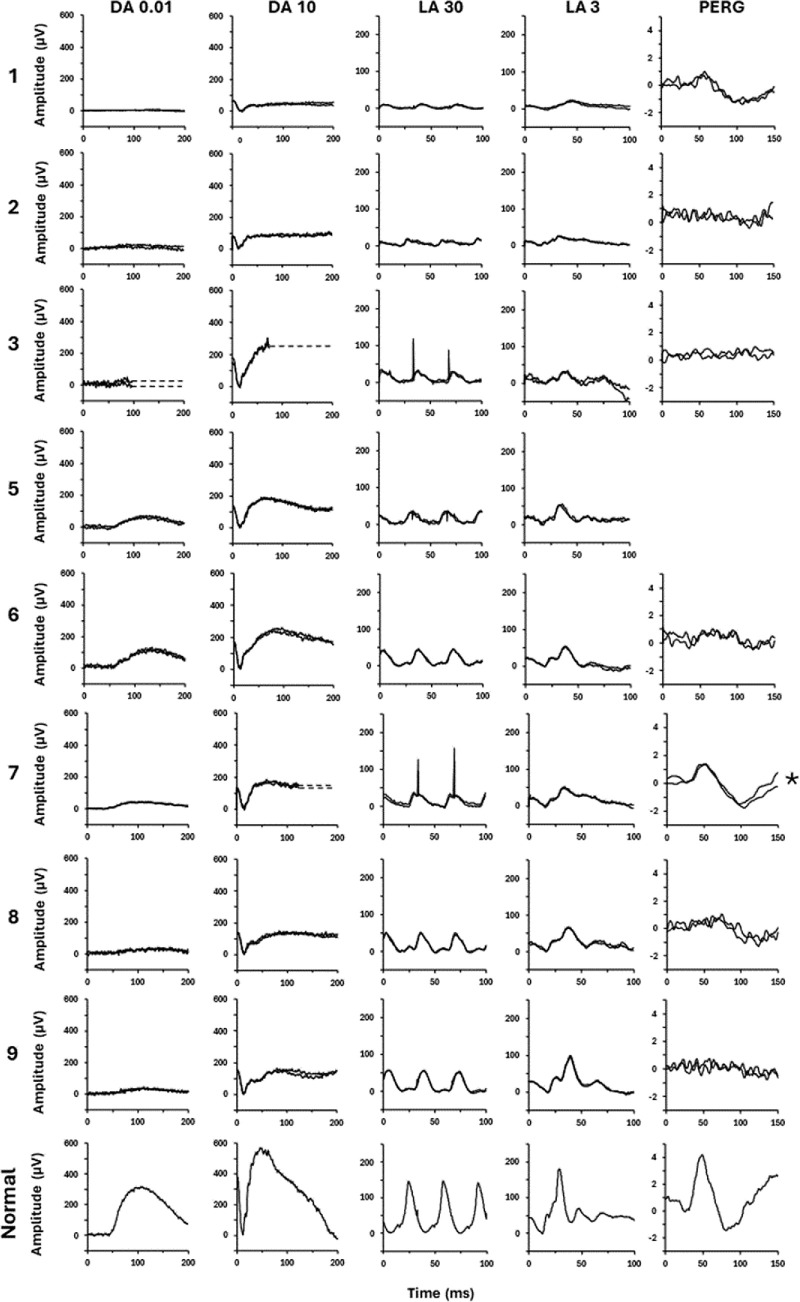
Representative ERG recordings for cases 1 to 3 and 5 to 9, tested according to ISCEV standard methods and from a representative control subject (N) for comparison. Patient waveforms are superimposed to demonstrate reproducibility. *Broken lines* replace blink and eye movement artifacts for clarity. For case 7 (*), a high-frequency electrical artifact was removed from the PERG by digital filtering, post-acquisition. Traces from case 4 are not shown (recorded with a different recording system).

The ISCEV full-field ERG findings are quantified in Figure 4, and examples of recordings are shown in Figure 5. The DA 0.01 and DA 10 ERG a-waves and b-waves were subnormal in nine of nine cases, and LA ERG components were subnormal in all but one individual, with a preserved LA 3 ERG b-wave (Fig. 4A, case 9). There was LA 30-Hz flicker delay in all nine cases (mean delay, 10 ms; range, 5–15 ms). The findings indicate generalized retinal dysfunction at the level of the photoreceptors, including two with a clear rod–cone pattern of dysfunction (cases 8 and 9) and others with a similar degree of rod and cone system involvement (cases 1–7). The LA 3 ERG b:a ratios were subnormal in five patients (cases 1, 3, 4, 6, and 7); the DA 10 ERG b:a ratios were additionally mildly subnormal in four patients (cases 1, 2, 8, and 9), including those with undetectable or severely reduced rod-system–specific DA 0.01 ERGs. DA 10 ERG b-waves showed delay in three patients (cases 6, 7, and 9). There was no obvious correlation between age and the severity of ERG abnormalities (Fig. 4A).

Six of nine cases had undetectable pattern ERG P50 components, consistent with severe macular involvement; P50 was detectable but subnormal in cases 1, 6, and 7, reduced by 55%, 65%, and 20%, respectively (Fig. 5).

Three individuals underwent follow-up recordings (Supplementary Fig. S2); two showed a high degree of ERG stability after 4 or 5 years, and one subject tested after 9 years had mildly increased LA 30-Hz peak times (by 3 ms), consistent with marginal worsening of cone system function. One of the two 5-year-old children tested with lower eyelid skin electrodes showed ERG evidence of a cone–rod dystrophy; the other showed mild 30-Hz flicker ERG delay, consistent with mild generalized cone system dysfunction. Undetectable pattern ERGs indicated macular involvement in both.

## Discussion

This study of *CRB1*-related retinal dystrophy reports detailed phenotyping of patients molecularly confirmed to have the p.(Pro836Thr) variant and characterizes their key clinical and electrophysiological features. The study extends previous investigations of *CRB1*-related retinopathy,^18^ highlighting the association of the p.(Pro836Thr) variant with African ancestry. The allele frequency of this variant is markedly different among various genetic ancestry groups; although it is observed at 0.329% in Africans/African Americans, it is absent in Europeans and Asians (gnomAD v4.1.0). Of note, research on IRDs has been comprehensively performed in Europe and North America, but a substantial proportion of patients remain genetically undiagnosed in Asia and Africa, which represent over 60% of the global population.^26^^,^^27^ The underrepresentation of specific racial/ethnic groups in population databases and disease–gene association databases makes variant interpretation more challenging and can lead to both over- and underdiagnosis of an inherited disorder.^28^^,^^29^ It is reasonable to infer that many causative IRD variants remain unknown, and expanding our knowledge of ethnic-specific variants is important to enhance diagnostic precision and support equitable health care.

Biallelic pathogenic variants in the *CRB1* gene are known to result in a diverse spectrum of retinopathies with phenotypic variability.^30^ Interestingly, although LCA is the most commonly reported phenotype for *CRB1*-associated retinal dystrophy (43%) and *CRB1* is implicated in 7% to 17% of autosomal recessive LCA cases, none of the patients in our cohort exhibited this phenotype.^6^^,^^31^ Literature review for this specific variant identified an additional 15 patients from nine publications, six of whom were homozygous for this variant.^9^^–^^12^^,^^16^^,^^32^^–^^36^ Altogether, 28 patients have an established genotype–phenotype correlation, and only one homozygous patient has been reported to exhibit the LCA phenotype.^33^ The unusual severity of this reported patient raises considerations of a hemizygous deletion, which may occur more commonly than recognized by currently utilized molecular diagnostic techniques.^37^ Additionally, none of the patients in our cohort had developed a Coats-like exudative vasculopathy after a median follow-up of 7 years, with a median age of 31 years at the last follow-up. Our study suggests that the p.(Pro836Thr) tends to confer a phenotype less severe than LCA, as the symptom onset and presentation occurred in mid- to late childhood, and the majority of patients did not have nystagmus, in contrast to vision loss and nystagmus in early infancy characteristic of LCA.^7^

In our cohort, there was considerable phenotypic diversity even among patients homozygous for the p.(Pro836Thr) variant, and between siblings with identical *CRB1* variants. The biological mechanisms driving this broad phenotypic variability are of scientific interest. An estimated 95% of multiexon genes generate multiple mRNA isoforms, and this could play an important role in modifying disease severity.^38^^,^^39^ *CRB1* has three isoforms found to be expressed at meaningful levels in the retina: *CRB1-A*, *CRB1-B*, and *CRB1-C*.^6^^,^^14^ Analysis of RNA isolated from retinas of donor eyes has revealed that *CRB1-B* is the predominant isoform in adult retina*.* Compared to the canonical isoform *CRB1-A*, there are differences at its 5′ and 3′ ends: Distinct promoters at the 5′ exon drive *CRB1-A* expression in Müller glial cells and *CRB1-B* expression in photoreceptors, whereas the 3′ exons encode different intracellular domains that may interact with different intracellular partners.^14^ Although limited genotype–phenotype correlations have been found for *CRB1*-linked IRDs, other than the association of the in-frame deletion p.(Ile167_Gly169) with a restricted macular dystrophy phenotype, null variants are associated with greater disease severity, and this is perhaps explained by a disruption of all *CRB1* isoforms.^17^^,^^19^ The p.(Pro836Thr) is predicted to affect all Müller cell and photoreceptor isoforms.^16^ This predicted effect is broadly consistent with the available electrophysiological findings in our patients showing generalized photoreceptor dysfunction, mostly with similar involvement of rods and cones, but with additional evidence of dysfunction post-phototransduction, or inner retinal, in six of nine cases. It was not possible to infer inner retinal dysfunction from the two patients with the most severe rod photoreceptor involvement (Fig. 4, cases 1 and 2), because in these two cases the reduced DA10 ERG b:a ratios may reflect the “photopic hill” phenomenon manifesting under conditions of dark adaptation, in the absence or near-absence of rod function, as has been described in some other conditions that selectively or predominantly affect the rod photoreceptors.^40^^–^^42^

In compound heterozygous patients, it remains to be determined how the second variants disrupt one or all isoforms of *CRB1* and how the degree of functional preservation in each isoform and level/ratio of the isoforms could collectively modify the disease severity and progression. An additional potential disease modifier stems from the presence of the *CRB1* gene paralog *CRB2* (OMIM 609720), which also encodes multiple protein isoforms.^2^^,^^6^^,^^43^ *CRB2* localizes to the (1) subapical region of Müller glial cells, (2) inner segment of photoreceptor inner segments, and (3) RPE cells.^6^^,^^44^^,^^45^ Another *CRB1* paralog, *CRB3*, localizes to the Müller glial cells, photoreceptors, rod bipolar cells, and vascular pericytes, but variants in this gene have yet to be linked to retinal disease.^4^^,^^46^^,^^47^ *CRB2* has been associated with autosomal recessive non-syndromic retinitis pigmentosa (RP).^48^ In mouse models, knockout of Crb1 was associated with an RP-like phenotype, but additional knockout of Crb2 gave rise to an LCA-like phenotype.^46^ Further, in Crb double-mutant mice, recombinant adeno-associated virus (rAAV)-mediated human *CRB2* gene delivery to Müller cells was demonstrated to partially preserve retinal morphology.^49^ Notably, expressing human *CRB1* in tissues lacking endogenous mouse Crb1 led to severe infiltration of immune cells, likely from a compromised blood–retinal barrier and immune privilege. However, expression of human *CRB2* did not trigger a similar immune response and may relate to ubiquitous expression in extraocular tissues reducing its immunogenicity.^47^ These preclinical studies provide preliminary evidence that *CRB2* gene therapy might have the potential to improve retinal function and morphology in *CRB1-*associated retinal dystrophy.^47^^,^^49^

In conclusion, the p.(Pro836Thr) variant is particularly prevalent in those of Black African ancestry. This variant is generally associated with a milder phenotype and is less likely to be associated with a severe LCA phenotype; however, electrophysiology in this series indicates that dysfunction is not restricted to the macula, as suggested in some but not all of the retinal imaging findings. Although the clinical variability remains incompletely understood, recent insights into the isoforms of *CRB1* suggest that post-transcriptional processes within Müller glial cells and photoreceptors are potential modifiers of disease severity.^16^ More work is needed to understand whether co-occurrence of variants in the gene paralog *CRB2* contributes to the mutation load and further disrupts retinal homeostasis.^47^ Further analysis of the *CRB1* isoforms expressed in patient-derived retinal organoids would be helpful to unravel the relevant molecular mechanisms and their possible impact on DNA- or RNA-editing therapeutic approaches.

## Supplementary Material

Supplement 1
