# Supplementary material for: A Phenotypic Study of CRB1 Retinopathy Secondary to the Variant p.(Pro836Thr) Prevalent in Those of Black African Ancestry
Source: Invest Ophthalmol Vis Sci. 2025 Jul 1;66(9):3. doi: 10.1167/iovs.66.9.3 (PMC12227024; doi:10.1167/iovs.66.9.3)
Supplement: Supplement 1 [file iovs-66-9-3_s001.pdf]

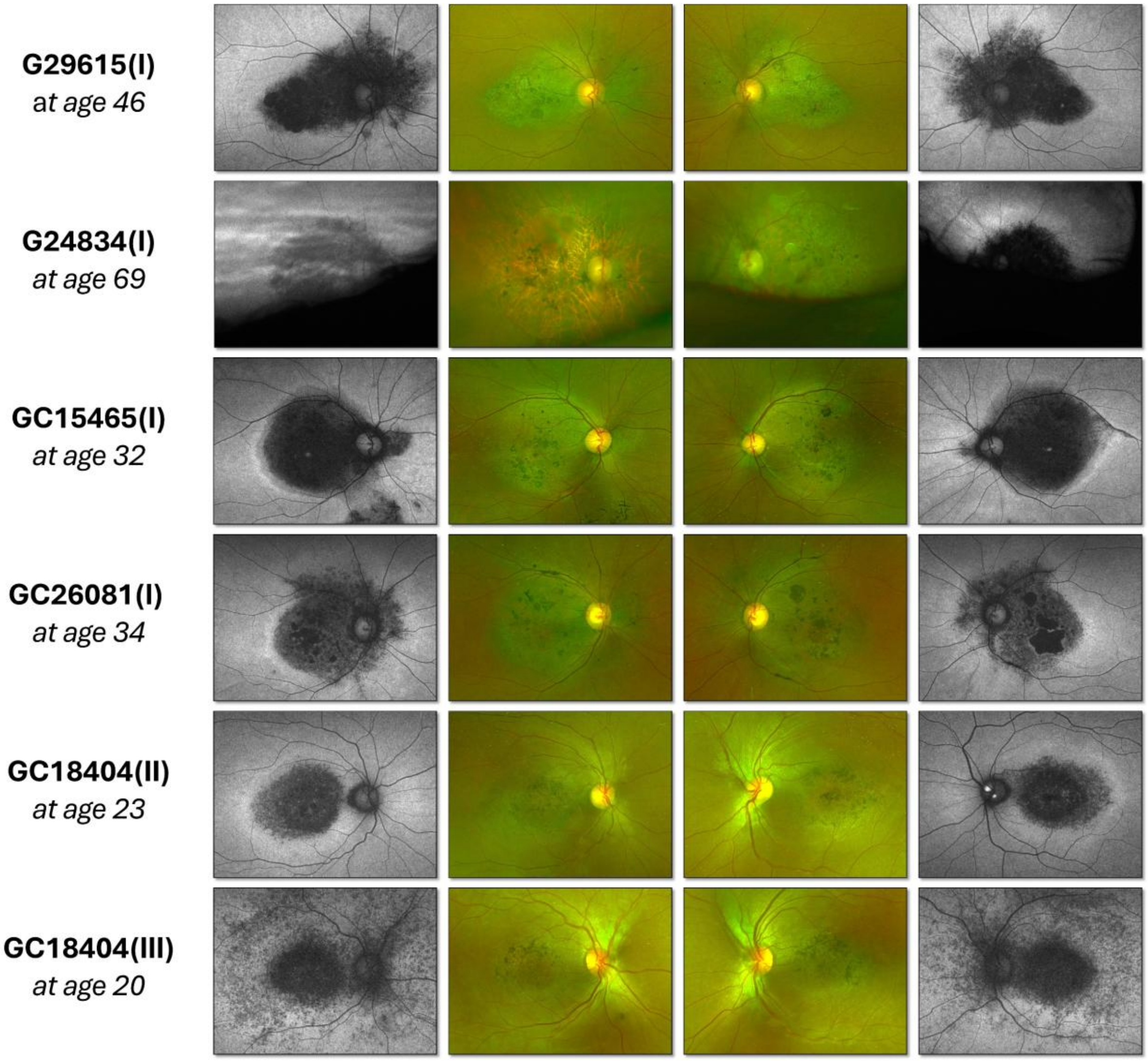

Supplementary Figure 1. Magnified view of the macular region on pseudocolour fundus photographs demonstrating nummular pigmentary changes in the macular region. The autofluorescence showed confluent hypoautofluorescence in the fovea, parafovea and perifoveal regions. Optic disc drusen was observed only in 1 eye [left eye of F11(I)].

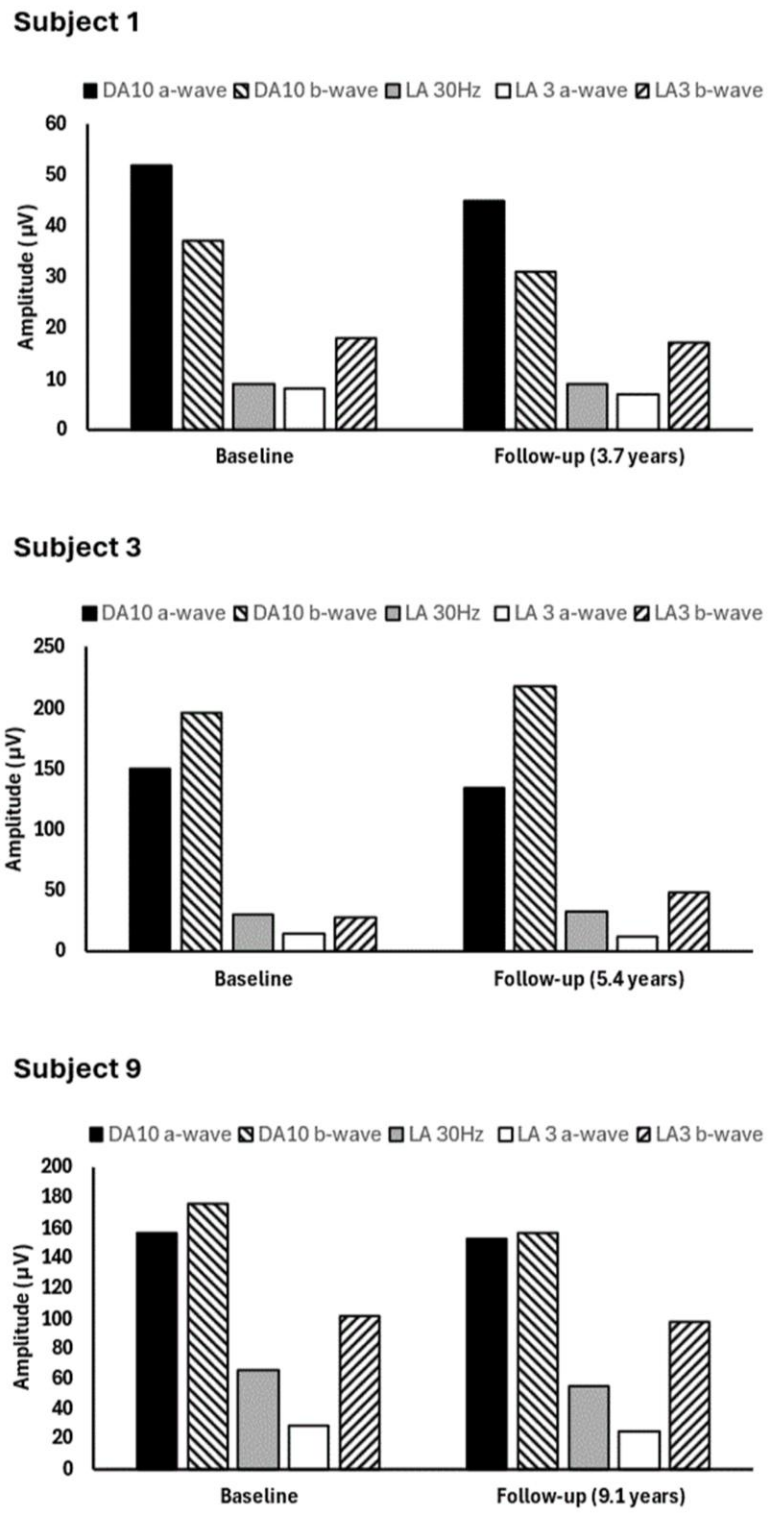

Supplementary Figure 2.

The main ERG component amplitudes compared in 3 subjects who underwent follow-up recordings after intervals of 3.7 years (subject 1), 5.4 years (subject 3), and 9.4 years (subject 9).
